# Differentiable Learning of Sequence-Specific Minimizer Schemes with DeepMinimizer

**DOI:** 10.1089/cmb.2022.0275

**Published:** 2022-12-13

**Authors:** Minh Hoang, Hongyu Zheng, Carl Kingsford

**Affiliations:** ^1^ Computer Science Department, and Carnegie Mellon University, Pittsburgh, Pennsylvania, USA.; ^2^ Computational Biology Department, Carnegie Mellon University, Pittsburgh, Pennsylvania, USA.

**Keywords:** deep learning, optimization, sequence sketching

## Abstract

Minimizers are widely used to sample representative *k*-mers from biological sequences in many applications, such as read mapping and taxonomy prediction. In most scenarios, having the minimizer scheme select as few *k*-mer positions as possible (i.e., having a low density) is desirable to reduce computation and memory cost. Despite the growing interest in minimizers, learning an effective scheme with optimal density is still an open question, as it requires solving an apparently challenging discrete optimization problem on the permutation space of *k*-mer orderings. Most existing schemes are designed to work well in expectation over random sequences, which have limited applicability to many practical tools. On the other hand, several methods have been proposed to construct minimizer schemes for a specific target sequence. These methods, however, only approximate the original objective with likewise discrete surrogate tasks that are not able to significantly improve the density performance. This article introduces the first continuous relaxation of the density minimizing objective, DeepMinimizer, which employs a novel Deep Learning twin architecture to simultaneously ensure both validity and performance of the minimizer scheme. Our surrogate objective is fully differentiable and, therefore, amenable to efficient gradient-based optimization using GPU computing. Finally, we demonstrate that DeepMinimizer discovers minimizer schemes that significantly outperform state-of-the-art constructions on human genomic sequences.

## 1. INTRODUCTION

Minimizers (Roberts et al, 2005; Schleimer et al, 2003) are *k*-mer sampling methods from a sequence such that sufficient information about the identity of the sequence is preserved. Minimizers are widely used to reduce memory consumption and run-time in bioinformatics applications such as genome assemblers (Ye et al, 2012), read mappers (Jain et al, 2022; Li, 2018), and *k*-mer counters (Deorowicz et al, 2015; Erbert et al, 2017).

Given a choice of *k*-mer length *k*, a window length *w*, and a total ordering π over all *k*-mers, a minimizer scheme selects the lowest priority *k*-mer from every overlapping window in the target sequence according to π. We typically measure minimizer performance by its density (Marçais et al, 2017) on a target sequence. Although alternative measures of performance are available (Edgar, 2021; Hach et al, 2012), this article will only focus on the density performance metric.

The choice of π has been known to significantly impact the resulting density on the target sequence. The theoretical lower-bound of density achievable by any minimizer scheme is given by O(1∕w) (Schleimer et al, 2003). On the other hand, a random initialization of π will yield an expected density of O(2∕w) (Marçais et al, 2017; Schleimer et al, 2003), which is frequently used as a baseline for comparing minimizer performance. This motivates the question: How do we effectively optimize π to improve the performance of minimizers?

Exhaustively searching the combinatorial space of π suffices for very small *k*, but it quickly becomes intractable for large values of *k* used in practice (e.g., k≥6) (Section 3.1). To work around this, many existing approaches focus on constructing minimizer schemes from mathematical objects with appealing properties such as universal hitting sets (UHS) (Ekim et al, 2020; Marçais et al, 2018; Marçais et al, 2017; Orenstein et al, 2017; Zheng et al, 2020). Although these schemes provide upper-bound guarantees for expected densities on random sequences, they only obtain modest improvements over a random minimizer when used to sketch a specific sequence (Zheng et al, 2020).

Learning minimizer schemes tailored toward a target sequence has been previously explored, although to a lesser extent. Current approaches include heuristic designs (Chikhi et al, 2016; Jain et al, 2020), greedy pruning (DeBlasio et al, 2019), and construction of *k*-mer sets that are well spread on the target sequence (Zheng et al, 2021). However, these methods only learn crude approximations of π by partitioning *k*-mers into disjoint subsets with different priorities to be selected. Within each partition, the relative ordering among *k*-mers depends on the choice of heuristic tie-breaking method (e.g., lexicographic or random). Hence, the resulting minimizer schemes are not necessarily optimal. We give a detailed overview of these methods in Section 2.

This article instead tackles the problem of directly learning a total order π. We note that the difficulty of this task comes from two factors, which we will review in detail in Section 3.1: (1) The search space of *k*-mer orderings is factorially large; and (2) the density minimizing objective is discrete. To overcome these challenges, we reformulate the original problem as parameter optimization of a deep learning system. This results in the first fully differentiable minimizer selection framework that can be efficiently optimized using gradient-based learning techniques. Specifically, our contributions are as follows:

1. We define a well-behaved search space for *k*-mer permutations that can efficiently leverage gradient-based optimization. This is achieved by representing *k*-mer orderings as continuous score assignments, output by a convolutional neural network called PriorityNet, whose architecture guarantees that any score assignment will correspond to a valid minimizer scheme (Section 3.2).
2. We then approximate the discrete density minimizing objective by a pair of surrogate sub-tasks: (a) generating valid minimizers; and (b) generating low density score assignments. As (a) is achieved by PriorityNet, we further design a complementary neural network called TemplateNet, which outputs potentially invalid assignments that are guaranteed to have low densities on the target sequence (Section 3.4). Minimizing the difference between the outputs of these networks using our proposed distance metric (Section 3.5) results in a valid consensus score assignment with low density. This results in the first fully differentiable objective (Section 3.3) for minimizer optimization.
3. Finally, we compare our framework, DeepMinimizer, against various state-of-the-art minimizer construction methods on human genomic data. We observe that DeepMinimizer yields sketches with significantly lower densities on various settings (Section 4) and obtains favorable running times through leveraging GPU computing.

## 2. RELATED WORK

### 2.1. UHS-based methods

Most existing minimizer selection schemes with performance guarantees over random sequences are based on the theory of UHS (Marçais et al, 2018; Orenstein et al, 2017). Particularly, a (w,k)-UHS is defined as a set of *k*-mers such that every window of length *w* (from any possible sequence) contains at least one of its elements. Every UHS subsequently defines a family of corresponding minimizer schemes whose expected densities on random sequences can be upper-bounded in terms of the UHS size (Marçais et al, 2017). As such, to obtain minimizers with provably low density, it suffices to construct small UHS, which is the common objective of many existing approaches (Ekim et al, 2020; Marçais et al, 2017; Zheng et al, 2020).

In the context of *sequence-specific* minimizers, there are several concerns with this approach. First, the requirement of UHS to “hit” all windows of *every possible* sequence is often too strong with respect to the need of sketching a specific string and results in sub-optimal UHS (Zheng et al, 2021). In addition, since real sequences rarely follow a uniform distribution (Zhang et al, 2007), there tends to be little correspondence between the provable upper-bound on expected density and the actual density measured on a target sequence. In practice, the latter is usually more pessimistic (Zheng et al, 2021; Zheng et al, 2020) on sequences of interest, such as the human reference genome, which drives the development of various *sequence-specific* minimizer selection methods.

### 2.2. Heuristic methods

Several minimizer construction schemes rank *k*-mers based on their frequencies in the target sequence (Chikhi et al, 2016; Jain et al, 2020), such that non-repetitive *k*-mers are more likely to be chosen as minimizers. These constructions, nonetheless, rely on the assumption that non-repetitive *k*-mers are spread apart and ideally correspond to a sparse sampling. Another greedy approach is to sequentially remove *k*-mers from an arbitrarily constructed UHS, as long as the resulting set still hits every *w*-long window on the target sequence (DeBlasio et al, 2019). Though this helps to fine-tune a given UHS with respect to the sequence of interest, there is no guarantee that such an initial set will yield the optimal solution after pruning.

### 2.3. Polar set construction

Recently, a novel class of minimizer constructions was proposed based on polar sets of *k*-mers, whose elements are sufficiently far apart on the target sequence (Zheng et al, 2021). The sketch size induced by such a polar set is shown to be tightly bounded with respect to its cardinality. This reveals an alternate route to low-density minimizer schemes through searching for the minimal polar set. Unfortunately, this proxy objective is NP-hard and currently approximated by a greedy construction (Zheng et al, 2021).

**Remark 1.** *In all of the above methods, the common objective to be optimized can be seen as a partition of the set of all k-mers into disjoint subsets. For example, frequency values are used to denote different buckets of k-mers* (Chikhi et al, 2016; Jain et al, 2020)*. Others* (DeBlasio et al, 2019; Ekim et al, 2020; Zheng et al, 2021; Zheng et al, 2020) *employ a more fine-grained partitioning scheme defined by the constructed UHS/polar set. Each subset has an assigned priority value, such that k-mers from higher priority subsets are always chosen over k-mers from lower priority subsets. However, it remains undetermined how k-mers from within the same subset can be optimally selected to recover a total ordering* π*. Practically, these methods resort to using a pre-determined arbitrary ordering to resolve such situations. In contrast, our work investigates a novel approach to directly learn this ordering.*

## 3. METHODS

### 3.1. Background

Let Σ be an alphabet of size |Σ|=σ and *S* be a sequence containing exactly *l* overlapping *k*-mers defined on this alphabet, that is, $S\in \Sigma^{l+k-1}$. For some $w\in {\mathbb{N}}^{+}$ such that l≥w, we define a (w,k)-window as a substring in *S* of length w+k−1, which contains exactly *w* overlapping *k*-mers. For ease of notation, we further let $l_{w}=\Delta l-w+1$ denote the number of (w,k)-windows in *S*. We will also assume that *w* and *k* are fixed and given as application-specific parameters.

**Definition 1. (Minimizer)** A minimizer scheme $m:\Sigma^{w+k-1}\to \left[1,w\right]$ is uniquely specified by a total ordering π on $\Sigma^{k}$. Here, we encode π as a function $\rho :\Sigma^{k}\to {\mathbb{N}}^{+}$ that maps *k*-mers to its position in π. Given a (w,k)-window ω, *m* then returns the smallest *k*-mer in ω according to ρ:

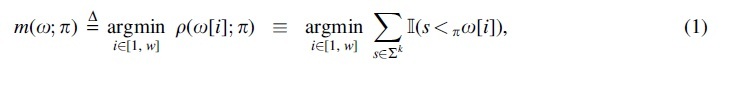

where ℐ denotes the indicator function, ω[i] denotes the *i*-th *k*-mer in ω, and $s<{}_{\pi }\omega \left[i\right]$ indicates *s* precedes ω[i] in π. We break ties by prioritizing *k*-mers that occur earlier in (i.e., to the left of) the window.

When applied to a sequence *S*, the scheme cited earlier selects one *k*-mer position from every overlapping window to construct the sequence sketch $ℒ\left(S;m\right)=\left\{t+m\left(\omega_{t}\right)|t\in \left[1,l_{w}\right]\right\}$, with $\omega_{t}$ denoting the tth window in *S*. Naturally, a smaller sketch leads to more space and cost savings. As such, we measure minimizer performance by the density factor metric $D\left(S;m\right)=\Delta \left|ℒ\left(S;m\right)\right|\times \left(w+1\right)∕l_{w}$, which approximates the number of *k*-mers selected per window (Marçais et al, 2017). The minimizer selection problem is then formalized as density minimization with respect to π:

(2) $$\pi^{*}={\mathrm{argmin}}_{\pi }D\left(S;m\left(\cdot ;\pi \right)\right)\equiv {\mathrm{argmin}}_{\pi }|ℒ\left(S;m\left(\cdot ;\pi \right)\right)|.$$

This objective, however, is intractable to optimize for two reasons. First, the number of all *k*-mer permutations scales super-exponentially with *k* and σ (i.e., $\sigma^{k}!$), thus rendering any form of exhaustive search on this space impossible under most practical settings. Further, the set counting operation |ℒ(S;m(⋅;π))| is non-differentiable even if the solution space is continuous, which makes efficient gradient-based optimizers inaccessible. The remainder of this section, therefore, proposes a deep-learning strategy to address both these challenges, and it is organized as follows.

Section 3.2 describes a unifying view of existing methods as reparameterizations of ρ (Definition 1). We then propose a novel deep parameterization called PriorityNet that relaxes the permutation search space of Equation (2) into a well-behaved weight space of a neural network. Section 3.3 shows that density optimization with respect to PriorityNet can be approximated by two sub-tasks via introducing another complementary network, called TemplateNet.

**FIG. 1.**
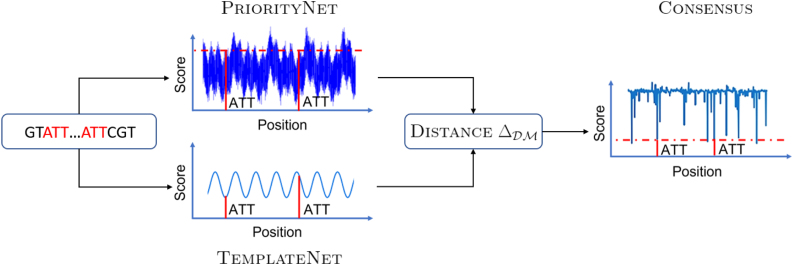
Our DeepMinimizer framework employs a twin network architecture. PriorityNet generates valid minimizers, but it has no guarantee on density. In contrast, TemplateNet generates low-density templates that might not correspond to valid minimizers. We minimize the distance between the outputs of these networks to obtain a consensus with low density on the target sequence.

This approximation can be formalized as a fully differentiable proxy objective that minimizes distance between TemplateNet and PriorityNet. Sections 3.4 and 3.5 then, respectively, discuss the parameterization of TemplateNet and the distance metric in our proxy objective, thus completing the specification of our framework (Fig. 1).

### 3.2. Search space reparameterization

We first remark that many existing methods can be seen as re-parameterizations of ρ in Definition 1. For example, ρ can be parameterized with frequency information from the target sequence (Chikhi et al, 2016; Jain et al, 2020), that is, $\rho \left(\omega_{i};S\right)\propto \sum_{j=1}^{l_{w}}ℐ\left(\omega_{j}=\omega_{i}\right)$; or instantiated with a UHS υ (Ekim et al, 2020; Zheng et al, 2020), that is, $\rho \left(\omega_{i};\upsilon \right)=ℐ\left(\omega_{i}⁄\in \upsilon \right)$. Similar set-ups have been explored in the context of sequence-specific minimizers using a pruned UHS υ(S) (DeBlasio et al, 2019) and a polar set ζ(S) (Zheng et al, 2021) constructed for the target sequence. Here, we note that the notation ρ is overloaded to admit different parameter representations. This is mainly to highlight the unification of existing methods, and it has no implication on the mathematical consistency of our formulation.

There are fewer discrete values potentially assigned by ρ than the total number of *k*-mers in all these re-parameterizations. As such, these methods still rely on a pre-determined arbitrary ordering to break ties in windows with two or more similarly scored *k*-mers. When collisions occur frequently, this could have an unexpected impact on the final density. DeepMinimizer instead employs a continuous parameterization of ρ using a feed-forward neural network parameterized by weights α, which takes as input the multi-hot encoding of a *k*-mer (i.e., a concatenation of its character one-hot encodings) and returns a real-valued score in [0,1].

This continuous scheme practically eliminates the chance for scoring collisions. Further, the solution space of this re-parameterization is only restricted by the modeling capacity encoded by our architecture weight space. This limitation quickly diminishes as we employ sufficiently large number of hidden layers in the network. We can subsequently rewrite Equation (2) as optimizing a neural network with density as its loss function:

(3) $$\alpha^{*}={\mathrm{argmin}}_{\alpha }D\left(S;\rho \left(\cdot ;\alpha \right)\right).$$

**FIG. 2.**
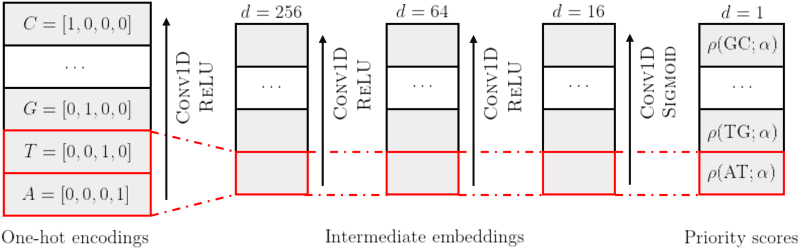
Our PriorityNet architecture for k=2, parameterized by weights α, maps sequence multi-hot encoding to priority scores through a series of three convolution layers with kernel size [k,1,1] and [256,64,16] embedding channels, respectively. Fixing network weights α, the computation of assigned priority score to any *k*-mer is deterministic given its character one-hot encodings.

Applying this network on every *k*-mer along *S* can be compactly written as a convolutional neural network, denoted by *f*, which maps the entire sequence *S* to a *score assignment* vector. We require this score assignment to be *consistent* across different windows to recover a valid ordering π from such implicitly encoded ρ. Specifically, one *k*-mer cannot be assigned different scores at different locations in *S*. To enforce this, we let the first convolution layer of our architecture, PriorityNet, have kernel size *k*, and all subsequent layers to have kernel size 1. An illustration for k=2 is given in Figure 2.

### 3.3. Proxy objective

The density computation in Equation (3), however, is not differentiable with respect to the network weights. As such, α cannot be readily optimized with established gradient back-propagation techniques used in most deep learning methods. To work around this, we introduce a proxy optimization objective that approximates Equation (3) via coupling PriorityNet with another function called TemplateNet. Unlike the former, TemplateNet relaxes the *consistency* requirement and generates *template* score assignments that might not correspond to valid minimizer schemes. In exchange, such *templates* are guaranteed to yield low densities by design.

Intuitively, the goals of these networks are complementary: PriorityNet generates valid minimizer schemes in the form of *consistent* priority score assignments, whereas TemplateNet pinpoints neighborhoods of low-density score assignments situated around its output templates. This reveals an alternative optimization route where these networks negotiate toward a consensus solution that (a) satisfies the constraint enforced by PriorityNet; and (b) resembles a template in the output space of TemplateNet, thus potentially yielding low density. Let *f* and *g* denote our proposed PriorityNet and TemplateNet, respectively parameterized by weights α and β; we formalize this objective as minimizing some distance metric Δ between their outputs:

(4) $$\left(\alpha_{*},\beta_{*}\right)={\mathrm{argmin}}_{\alpha ,\beta }\Delta \left(f\left(S;\alpha \right),g\left(S;\beta \right)\right).$$

In the remainder of this article, we detail the full specification of our proxy objective, which requires two other ingredients. First, Section 3.4 discusses the parameterization of our TemplateNet *g* to consistently generate templates that achieve the theoretical lower-bound density (Marçais et al, 2017) on the target sequence. Further, we note that the proxy objective in Equation (4) will perform best when the distance metric Δ reflects the difference in densities of two score assignments.

Section 3.5 then discusses a practical choice of Δ to accurately capture high-performing neighborhoods of minimizers. These specifications have strong implications on the expressiveness of the solution space and directly influences the performance of our framework, as shown in Section 4.

### 3.4. Specification of TemplateNet

The well-known theoretical lower bound 1+1∕w for density factor (Marçais et al, 2017) implies that the optimal minimizer, if it exists, samples *k*-mers exactly *w* positions apart. As a result, we want to construct TemplateNet such that its output approximates this uniform assignment pattern given any initialization of its parameter β. To obtain this construction, we first impose that our TemplateNet function 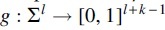 is specified by a continuous function 
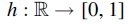
 such that 
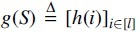
 concatenates evaluations of *h* at integer indices (i.e., *k*-mer positions). Then, Proposition 1 given next shows a sufficient construction of *h* such that g(S) approximately yields the optimal density.

**Proposition 1.** *Let* h:ℛ→[0,1] *be a periodic function, with fundamental period w, such that h has a unique minimum value on every w-long interval. Formally, h satisfies:*

(1):∀t∈ℛ:h(t)=h(t+w),and

$$\left(2\right):\forall i,j\in {\mathrm{arginf}}_{t}h\left(t\right),\exists u\in \mathbb{N}:|i-j|=uw.$$

*Then, the template* 
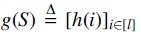
 *defined by h induces a sketch with density factor* 1+1∕w+o(1) *on S when S is sufficiently long (*i.e., $l_{w}\gg w^{2}$).

*Proof.* We first re-express the density factor of *S* in terms of the template score assignment g(S). Note that this expression will hold for a general continuous score vector and is appropriate regardless of whether g(S) satisfies the consistency constraint (Section 3.2). Let mt =Δ argminj∈ωth(j) be the minimum index of window $\omega_{t}$, and let $\gamma_{t}$ indicate the event that the *t*-th window picks a different *k*-mer than the (t−1)-th window. Particularly, $\gamma_{1}=\Delta 1$ and $\gamma_{t}=\Delta ℐ\left(m_{t}\neq m_{t-1}\right)$. Then, the density factor of the minimizer scheme induced by g(S) is given by:

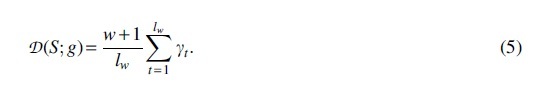

For any value of $u\in {\mathbb{N}}^{+}$, we further define the integer interval ${ℐ}_{u}=\Delta \left[\left(u-1\right)w+1,uw\right]$. As the density of the entire sequence is simply the sum of density for each interval ${ℐ}_{u}$, it is then sufficient to derive the values of $\gamma_{t}$ for all values of *t* in some arbitrary interval ${ℐ}_{u}$.

Without loss of generality, we assume $0\in {\mathrm{arginf}}_{t}h\left(t\right)$ since this can always be achieved via adding a constant phase shift to *h*. As *h* has a period of *w*, this implies $\left\{uw|u\in {\mathbb{N}}^{+}\right\}\subseteq {\mathrm{arginf}}_{t}h\left(t\right)$, which further reduces to $\left\{uw|u\in {\mathbb{N}}^{+}\right\}\equiv {\mathrm{arginf}}_{t}h\left(t\right)$ when condition (2) holds. Then, it follows that ∀t≠uw, we have $t\notin {\mathrm{arginf}}_{t}h\left(t\right)$. In addition, we also have $uw\in \omega_{t}$ by definition of $\omega_{t}$. Together, the facts cited earlier imply that $\forall t:m_{t}=uw$ and consequently $\gamma_{t}=0$ for all t≠(u−1)w+1, since $uw\notin \omega_{\left(u-1\right)w+1}$.

For u=1, we trivially have $\gamma_{\left(u-1\right)w+1}=\gamma_{1}=1$ by definition. For any u>1, we have $m_{\left(u-1\right)w}=\left(u-1\right)w$, and $m_{\left(u-1\right)w}+1=uw$, which imply $\gamma_{\left(u-1\right)w+1}=1$. Finally, using the earlier cited derivations, we have:

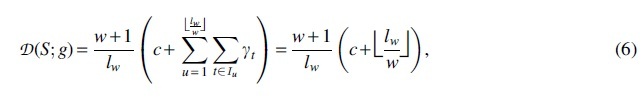

where $c=\Delta \sum_{t=\left\lfloor \frac{l_{w}}{w}\right\rfloor w+1}^{l_{w}}\gamma_{t}$ is the remainder of the sequence that does not make up any complete interval. The second equality follows from the derived values of $\gamma_{t}$ for $t\in {ℐ}_{u}$. Finally, using the fact that $c=l_{w}-\left\lfloor \frac{l_{w}}{w}\right\rfloor w<w$ and the sufficient length assumption $l_{w}\gg w^{2}$, we have:

(7) $$\frac{w+1}{l_{w}}\left(c+\left\lfloor \frac{l_{w}}{w}\right\rfloor \right)<\frac{w+1}{w}+\frac{w\left(w+1\right)}{l_{w}}=1+\frac{1}{w}+o\left(1\right),$$

which concludes our proof.□

Note that the resulting sketch induced by *h* does not necessarily correspond to a valid minimizer. Although this sketch has guaranteed low density, it does not preserve the sequence identity like a minimizer sketch; hence, it is not useful for downstream applications. However, it is sufficient as a guiding template to help PriorityNet navigating the space of orderings.

**Remark 2.** *By Proposition 1*, TemplateNet *can be as simple as* h(t)=sin(2πt∕w) *to induce a near-optimal score assignment. This naive specification, however, encodes exactly a single set of template minima (*i.e., *one that picks k-mers from the set of interval positions* {w,2w,…}*), which might not be in proximity of any valid minimizer scheme. For example, consider a sequence S in which some particular k-mer uniquely occurs at positions* $t\in \left\{\frac{1}{2}w,\frac{3}{2}w,\ldots \right\}$*. The ideal assignment would then be for the template minima to occur at these locations, which is not possible given the above choice of h*.

It is, therefore, necessary that the specification of TemplateNet is sufficiently expressive for Equation (4) to find an optimal solution. To model this family of template functions, we subsequently propose several parameterization strategies using (1) an ensemble of sinusoidal functions with integer phase shifts or (2) a Fourier series model that encodes any arbitrary sinusoidal function. We further propose an independent positional phase-delay component that can be combined with (1) and (2) to encode template functions with an approximately constant period.

#### 3.4.1. Ensemble template model

This section gives a construction of a periodic model such that every *k*-mer position appears in at least one template encoded by its parameter space. To achieve this, we employ a linear combination of multiple sine functions with fixed integer phase shifts ϕ∈[w−1], each of which encodes a set of minima with a unique positional offset such as $T_{1}=\left\{0,w,2w,\ldots \right\},T_{2}=\left\{1,w+1,2w+1,\ldots \right\},\ldots T_{w-1}=\left\{w-1,2w-1,3w-1,\ldots \right\}$. In particular, we define:


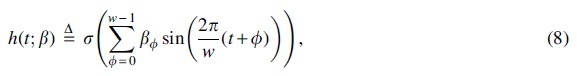


where the sigmoid activation function σ ensures that h(t) appropriately maps to [0,1] and outputs scores on the same scale as PriorityNet; $\beta ={\left\{\beta_{\phi }\right\}}_{\phi =0}^{w-1}$ are optimizable amplitude parameters such that $\beta_{\phi }\geq 0$ and $\sum_{\phi =1}^{w}\beta_{\phi }=1$. Optimizing β then determines the dominant phase shift $\phi_{max}=argmax_{\phi }\beta_{\phi }$, which, in turn, controls the final offset of the template minima. In addition, by allowing the amplitudes of the ensemble components to be optimizable, we also generate sufficient slack room such that the template scores can be accurately matched against the priority scores.

#### 3.4.2. Truncated Fourier series template model

A more general way to encode any periodic function h(t) with period *w* is via using a Fourier series that linearly combines an infinite number of sine and cosine functions whose frequencies are integer multiples of 1∕w:


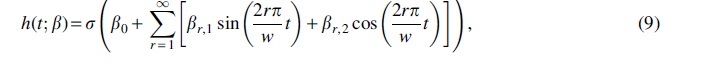


where $\beta ={\left\{\beta_{r,1},\beta_{r,2}\right\}}_{r=0}^{\infty }\cup \left\{\beta_{0}\right\}$ are optimizable amplitude parameters. For computational efficiency, we approximate this template model by a finite truncation up to the first *R* summands of the Fourier series cited earlier:


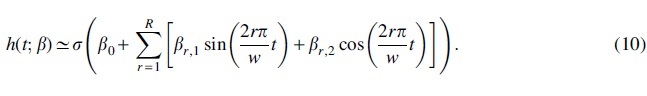


Similar to the ensemble template model, optimizing the amplitude parameters β of this model also determines the offset of the minima locations and adds slack room to help matching against the priority score assignment. The key difference between these two template models is that the ensemble model requires all *w* phase shifts (and hence, all *w* component functions) to encode every *k*-mer location, whereas the Fourier model can achieve the same with a fixed value of *R* and remains compact even for large *w*.

The Fourier model, however, will admit periodic functions whose minima do not coincide with integer indices; therefore, condition (2) cited earlier will be less likely to hold in practice. We will explore this trade-off in our empirical study given next (Section 4).

#### 3.4.3. Positional phase-shift model

By Proposition 1, all template score assignments encoded by the earlier cited β-parameterized families of functions correspond to near-optimal minimizer schemes with approximately perfect density factors. However, we note that this set of template solutions is usually unrealistic and cannot be mirrored exactly by PriorityNet, especially on complex problem instances with more difficult scoring constraints. For example, although the theoretical lower bound for density factor is 1+1∕w, the actual optimal density factor attainable given a specific sequence is often considerably larger and occurs when consecutive minimizer locations are not always exactly *w* locations apart.

Motivated by this observation, we further extend our template model with a learnable component that adaptively adjusts the local frequencies of every encoded periodic function through adding positional noise to their phase-shift parameters. That is, let 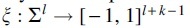 be a noise generating function parameterized by γ and let ξ(S;γ) be the output noise vector corresponding to the input sequence *S*. We define the δ-augmented template function as:

(11) $$g\left(S;\beta ,\gamma \right)=\Delta {\left[h\left(i+\delta \cdot \xi_{i}\left(S;\gamma \right);\beta \right)\right]}_{i\in \left[l\right]},$$

where $\xi_{i}\left(S;\gamma \right)$ denotes the *i*-th entry of the noise vector. This will allow every entry in the template score assignment to be adjusted by a phase shift of up to δ in magnitude. When δ=0, this space of template functions coincides with that of the exact periodic template model, thus encoding all theoretical optimal assignments. On the other hand, as δ increases, more template assignments are admitted, but the optimal density guarantee becomes less certain. We will explore the effect of this trade-off in Section 4.

### 3.5. Specification of the distance metric

As standard practice, we first consider instantiating with the $\ell^{2}$ distance. For ease of notation, let f =Δf(S;α) and g =Δg(S;β,γ), respectively, denote the score assignments output by PriorityNet and TemplateNet given *S*; then, 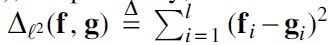. This metric, however, places an excessively strict matching objective at all locations along f and g. Such perfect matching is often unnecessary, as long as the *k*-mers outside selected locations (by the induced minimizer scheme) are assigned higher scores. In fact, enforcing a perfect matching will only take away the degrees of freedom needed for the proxy objective to satisfy the constraints implied by PriorityNet.

As such, we are interested in constructing an alternative distance metric that: (a) prioritizes matching f and g around the neighborhoods of minima; and (b) allows flexible assignment at other positions to admit more solutions that meet the consistency requirement. To accomplish these design goals, we propose the following asymmetrical distance metric:


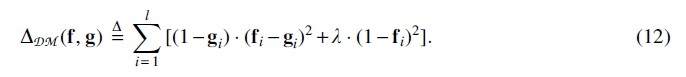


Specifically, the intuition behind the first component 
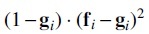
 in the summation is to weight each position-wise matching term 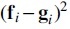 by its corresponding template score: The weight term $1-g_{i}$ implies stronger matching preference around the minima of g where the template scores $g_{i}$ are low; and vice-versa weaker matching preference at other locations where $g_{i}$ are high. The second component 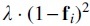, on the other hand, encourages PriorityNet to maximize its output scores whenever possible, which prevents the system from settling for a trivial solution where both f and g are squashed to zero.

The trade-off between these two components is controlled by the magnitude of the hyper-parameter λ. Finally, we confirm that this distance metric is fully differentiable with respect to α,β, and γ; hence, it can be efficiently optimized using gradient-based techniques. Particularly, the parameter gradients of both networks are given by:

$$\frac{\partial }{\partial \alpha }\Delta_{Dℳ}\left(f,g\right)=\sum_{i=1}^{l}a_{i}\cdot \frac{\partial }{\partial \alpha }f_{i},$$

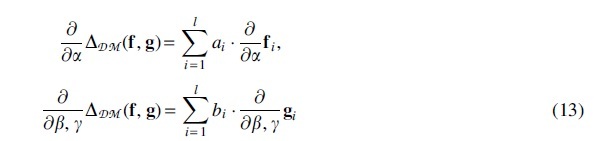


where the partial derivatives of network outputs are obtained via back-propagation and their respective constants are given by:

$$a_{i}=2\cdot \left(1-g_{i}\right)\cdot \left(f_{i}-g_{i}\right)+2\lambda \cdot \left(f_{i}-1\right),$$

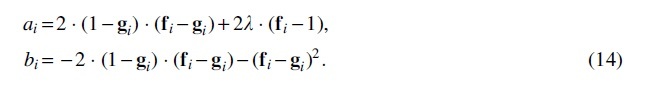


## 4. RESULTS

### 4.1. Implementation details

We implement our method using PyTorch and deploy all experiments on a RTX-2060 GPU. Due to limited GPU memory, each training epoch only computes a batch loss that averages over N=10 randomly sampled subsequences of length l=500×(w+k). We set λ=1 and use architectures of PriorityNet and TemplateNet as given in Figure 2 and Section 3.4, respectively. Network weights are optimized using the ADAM optimizer (Kingma and Ba, 2015) with learning rate $\eta =5e^{-3}$. Our implementation is available at https://github.com/Kingsford-Group/deepminimizer.

### 4.2. Comparison baselines

We compare DeepMinimizer with the following benchmarks: (a) random minimizer baseline; (b) Miniception (Zheng et al, 2020); (c) PASHA (Ekim et al, 2020); and (d) PolarSet Minimizer (Zheng et al, 2021). Among these methods, (d) is a sequence-specific minimizer scheme. For each method, we measure the density factor D obtained on different segments of the human reference genome: (a) chromosome 1 (Chr1); (b) chromosome X (ChrX); (c) the centromere region of chromosome X (Miga et al, 2020) (which we denote by ChrXC); and (d) the full genome (Hg38).

We used lexicographic ordering for PASHA as suggested by Zheng et al. (2020). Random ordering is used to rank *k*-mers within the UHS for Miniception, and outside the layered sets for PolarSet. In most settings, we employ the Ensemble template model (Section 3.4.1) with no positional phase-shift component (Section 3.4.3) for DeepMinimizer. However, for scenarios with large *w* values, we demonstrate that the Fourier template model with positional phase-shift is able to achieve better performance (Section 4.8)

### 4.3. Visualizing the mechanism of DeepMinimizer

**FIG. 3.**
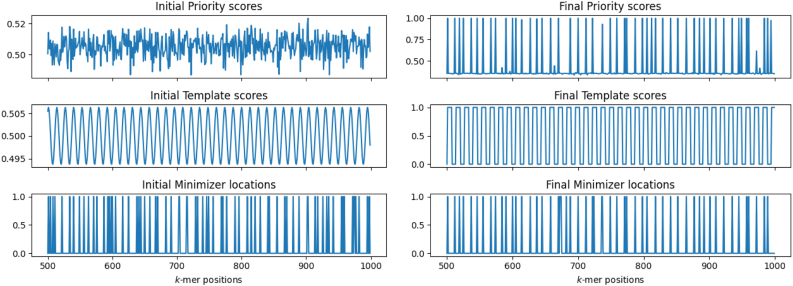
Visualization of PriorityNet and TemplateNet score assignments on positions 500−1000 of ChrXC with w=13, k=8. Left: Initial assignments (D=2.05); Right: Final assignments after 600 training epochs (D=1.39). The bottom plots show corresponding locations of sampled *k*-mers: a value of 1 means selected, and 0 otherwise.

First, we show the transformation of the priority scores assigned by ScoreNet and TemplateNet over 600 training epochs. Figure 3 plots the outputs of these networks evaluated on positions 500 to 1000 of ChrXC, and their corresponding locations of sampled *k*-mers.

**Remarks 3.** *For ease of implementation, we employ the standard* MaxPool *operator from PyTorch to select window maxima as minimizer locations (instead of window minima, as previously formulated). As a result, we expect the sampled locations in* Figure 3 *to coincide with the peaks of the priority scores (instead of the troughs). We also note that to accommodate this implementation, every relevant term in the* DeepMinimizer *objective has been properly negated.*

Initially, the PriorityNet assignment resembles that of a random minimizer and expectedly yields D=2.05. After 600 training epochs, the final TemplateNet assignment converges with a different phase shift than its initial assignment, but its period remains the same. Simultaneously, PriorityNet learns to match this template, hence it induces a visibly sparser sketch with D=1.39. This result demonstrates the negotiating behavior of our twin architecture to find optimal consensus score assignments.

### 4.4. Convergence of our proxy objective

**FIG. 4.**
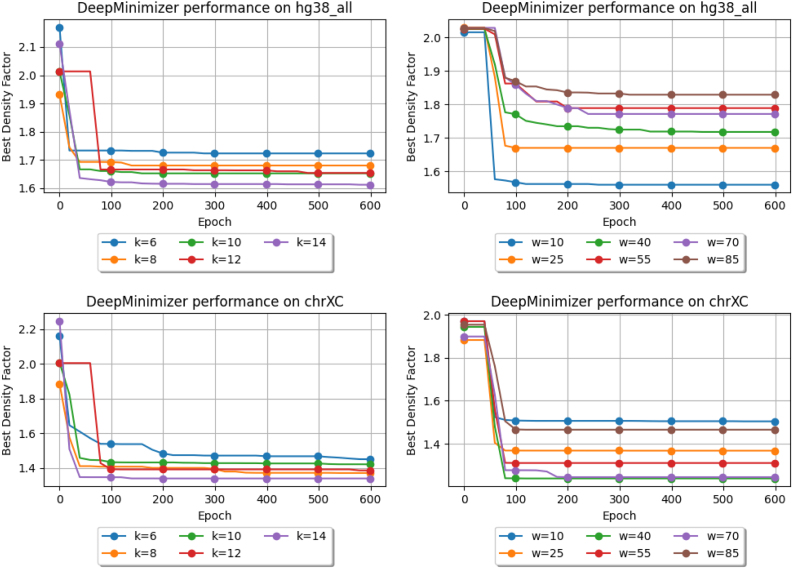
Best density factors obtained by DeepMinimizer on Hg38, ChrXC over 600 training epochs. Left: fix w=13, and vary k∈{6,8,10,12,14}; Right: fix k=14, and vary w∈{10,25,40,55,70,85}.

We further demonstrate that our proxy objective meaningfully improves minimizer performance as it is optimized. The first two columns of Figure 4 show the best density factors achieved by our method over 600 epochs on two scenarios: (a) varying *k* with fixed *w*; and (b) varying *w* with fixed *k*. The experiment is repeated on ChrXC and Hg38. In every scenario, DeepMinimizer starts with D≃2.0, which is only comparable to a random minimizer. We observe a steady decrease of D over the first 300 epochs before reaching convergence, where total reduction ranges from 11% to 23%.

Generally, larger *k* values lead to better performance improvement at convergence. This is expected since longer *k*-mers are more likely to occur uniquely in the target sequence, which makes it easier for a minimizer to achieve sparse sampling. In fact, previous results have shown that when *k* is much smaller than logw, no minimizer will be able to achieve the theoretical lower-bound D (Zheng et al, 2020). On the other hand, larger *w* values lead to smaller improvements and generally slower convergence. This is because our ensemble parameterization of TemplateNet scales with the window size *w* and becomes more complicated to optimize as *w* increases.

### 4.5. Evaluating our proposed distance metric

**FIG. 5.**
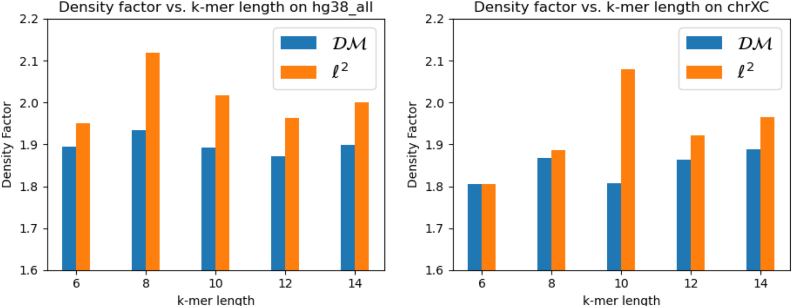
Comparing best density factors obtained by DeepMinimizer with $\Delta_{\ell^{2}}$ and $\Delta_{Dℳ}$ on Hg38 (left) and ChrXC (right) over 600 training epochs.

Figure 5 shows the density factors achieved by our DeepMinimizer method, respectively specified by the proposed distance metric $\Delta_{Dℳ}$ in Equation (12) and $\Delta_{\ell^{2}}$ distance. Here, we fix w=13 and vary k∈{6,8,10,12,14}. We observe that with the $\Delta_{\ell^{2}}$ distance, we obtain performance similar to a random minimizer in most cases. On the other hand, with our divergence function, DeepMinimizer obtains significantly lower densities, which confirms the intuition in Section 3.5.

### 4.6. Comparing against other minimizer methods

**FIG. 6.**
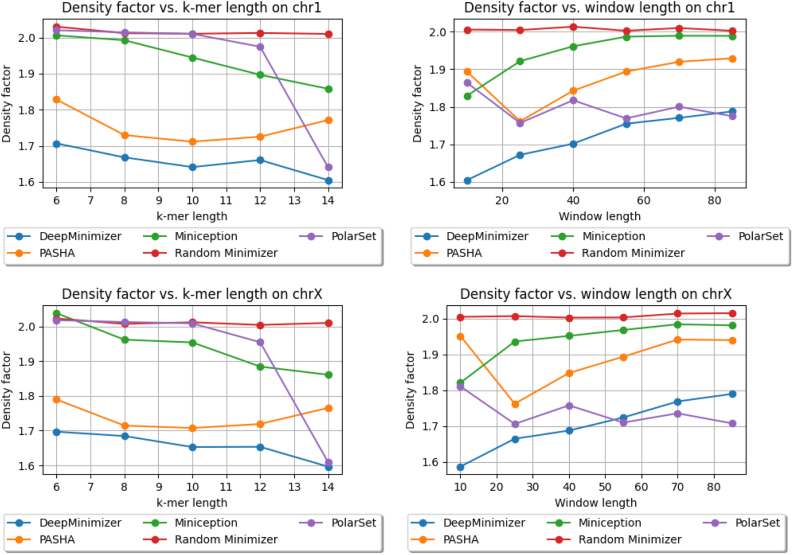
Density factors obtained by DeepMinimizer (600 training epochs), Random Minimizer, PASHA, Miniception and PolarSet on Chr1, ChrX. Left: fix w=13, and vary k∈{6,8,10,12,14}; Right: fix k=14, and vary w∈{10,25,40,55,70,85}.

**FIG. 7.**
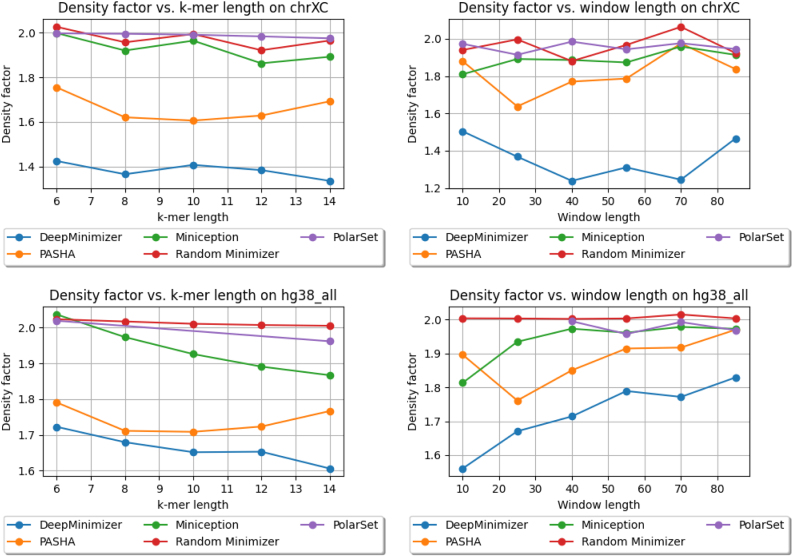
Density factors obtained by DeepMinimizer (600 training epochs), Random Minimizer, PASHA, Miniception and PolarSet on ChrXC, Hg38. Left: fix w=13, and vary k∈{6,8,10,12,14}; Right: fix k=14, and vary w∈{10,25,40,55,70,85}.

We show the performance of DeepMinimizer compared with other benchmark methods. In this experiment, DeepMinimizer is trained for 600 epochs with ensemble TemplateNet and no positional phase-shift. Figures 6 and 7 show the final density factors achieved by all methods, again on two comparison scenarios: (a) fix w=13, and vary k∈{6,8,10,12,14}; and (b) fix k=14, and vary w∈{10,25,40,55,70,85}. DeepMinimizer consistently achieves better performance compared with *non-sequence-specific* minimizers (i.e., PASHA, Miniception) on all settings.

We observe up to 40% reduction of density factor (e.g., on ChrXC, w=70, k=14), which clearly demonstrates the ability of DeepMinimizer to exploit *sequence-specific* information. Further, we also observe that DeepMinimizer outperforms our *sequence-specific* competitor, PolarSet, in a majority of settings. The improvements over PolarSet are especially pronounced for smaller *k* values, which are known harder tasks for minimizers (Zheng et al, 2020). On larger *w* values, our method performs slightly worse than PolarSet in some settings. This is likely due to the added complexity of optimizing TemplateNet, as described in the convergence ablation study of our method.

Notably, the centromere region of chromosome X (i.e., ChrXC) contains highly repetitive subsequences (Fukagawa and Earnshaw, 2014) and has been shown to hamper performance of PolarSet (Zheng et al, 2021). Figure 7 shows that PolarSet and the UHS-based methods perform similarly to a random minimizer, whereas our method is consistently better. Moreover, we observe that DeepMinimizer obtains near-optimal densities with ChrXC on several settings. For example, we achieved D=1.22 when k=14, w∈{40,70}, which is significantly better than the results on Chr1 and ChrX. This suggests that ChrXC is not necessarily more difficult to sketch, but rather good sketches have been excluded by the UHS and polar set reparameterizations, which is not the case with our framework.

### 4.7. Number of unique *k*-mers in the final minimizer set

**FIG. 8.**
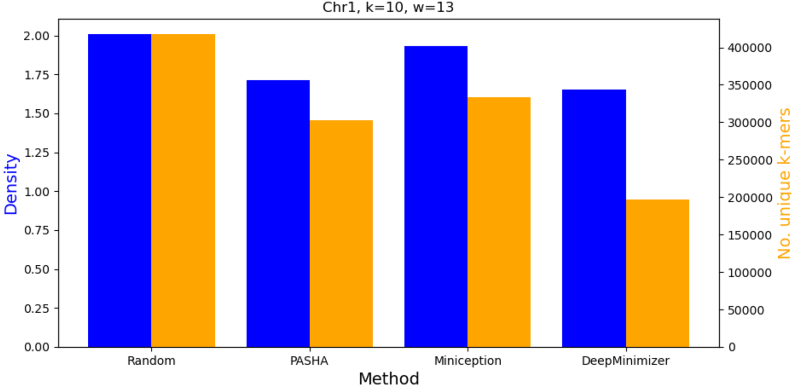
Comparing density and number of unique *k*-mers in the minimizer sets obtained by various benchmarks on Chr1 with k=10 and w=13.

This section investigates the numbers of unique *k*-mers in the final minimizer sets obtained by random ordering, PASHA, Miniception, and DeepMinimizer. On Chromosome 1, with k=10 and w=13, Figure 8 shows that the density factors and numbers of unique *k*-mers obtained by each method are strongly correlated. This agrees with the intuition of many other minimizer methods that a small set of high priority *k*-mers (e.g., a small UHS in the case of PASHA and Miniception) tends to induce a low-density sketch on the target sequence. This observation is also expected since the 10-mer distribution of Chr1 is fairly similar to that of a random sequence, which aligns with the premise of most UHS-based minimizer theories.

**FIG. 9.**
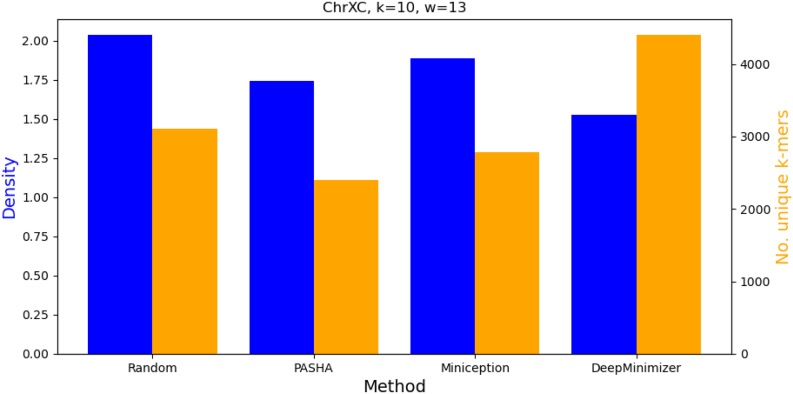
Comparing density and number of unique *k*-mers in the minimizer sets obtained by various benchmarks on ChrXC with k=10 and w=13.

However, on the chromosome region of ChrX, which contains many highly repetitive sub-sequences, Figure 9 shows that to achieve the best density (i.e., D=1.526), DeepMinimizer actually had to pick more high priority *k*-mers, not fewer. This interestingly demonstrates that minimizing the size of the UHS is not always a desirable surrogate objective on certain specific sequences, hence it asserts the need for a robust sequence-specific optimizer.

### 4.8. Comparing template models on large window values

In this section, we investigate the performance of DeepMinimizer on a large window size with different template models. Particularly, we fixed k=20,w=100 and compare the best density factor obtained by DeepMinimizer over 1200 training epochs using the ensemble template model (Section 3.4.1) and the truncated Fourier series template model (Section 3.4.2). We further pair each template model with a positional phase-shift component (Section 3.4.3), with δ∈{0.0,1.0,10.0}. We note that in each case, δ=0.0 corresponds to the original template model.

**FIG. 10.**
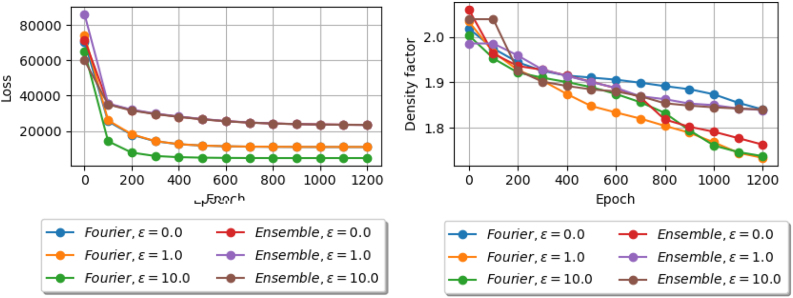
Comparing loss (left) and best density obtained (right) over 1200 training epochs on Chr1 between ensemble and truncated Fourier series template models. Each template model is paired with a positional phase-shift component with δ∈{0.0,1.0,10.0}.

Figure 10 shows the respective loss and density factor over 1200 training epochs of these template models. First, we observe that in all models, the loss values correlate positively with the corresponding density factor. Generally, as the DeepMinimizer loss decreases, the induced minimizer scheme also yields lower density factor on the input sequence, which suggests that our loss function is a good surrogate for the discrete density objective.

Further, we observe that among variants of the Fourier template model, both δ=1.0 and δ=10.0 perform significantly better than δ=0.0. This is most likely because adding local phase perturbations, indeed, allows TemplateNet to encode more realistic near-optimal score assignments. In contrast, among variants of the ensemble template model, δ=0.0 performs the best. This is most likely because the ensemble model has already accounted for all possible integer phase-shifts. As such, adding noisy phase perturbations with a magnitude greater than 1.0 will negatively affect the convergence of DeepMinimizer.

Finally, pairing Fourier template model with a positional phase-shift component of magnitude δ=1.0 achieves the best performance out of all variants. This aligns with our intuition in Section 3.4.2 regarding the trade-off between the certainty of Proposition 1 and the expressiveness of the admitted set of template score assignments.

### 4.9. Runtime performance

Finally, we confirm that DeepMinimizer runs efficiently with GPU computing. In all of our experiments, each training epoch takes ∼30 seconds to 2 minutes, depending on the choice of *k* and *w*, which controls the batch size. Performance evaluation takes between several minutes (ChrXC) to 1 hour (Hg38), depending on the length of the target sequence. Generally, our method is cost-efficient without frequent evaluations. Our most cost-intensive experiment (i.e., convergence ablation study on Hg38) requires a full-sequence evaluation every 20 epochs over 600 epochs, thus it takes ∼2 days to complete.

This is faster than PolarSet, which has a theoretical runtime of $O\left(n^{2}\right)$ and takes several days to run with Hg38. We note that in real applications, we only have to evaluate once by the end of the training loop, which is much faster compared with PolarSet, whose running time above only involves building the minimizer scheme.

**FIG. 11.**
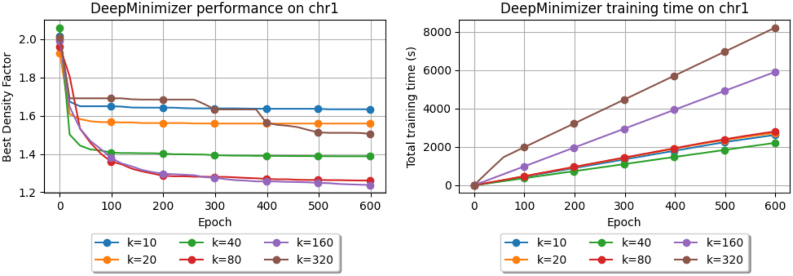
Best density obtained (left) and runtime (right) of DeepMinimizer for w=13 and k∈{10,20,40,80,160,320} on Chr1.

Figure 11 (right) measures runtime (in seconds) of DeepMinimizer on Chr1 over 600 epochs. Larger *k* values require PriorityNet to have more parameters. We expect running time for k=40,80,160,320 to increase in the same order. For k=10 and 20, however, the running times are approximately the same as k=80. We note that a smaller *k* value means there are more *k*-mers in the same sequence. As such, even though PriorityNet is more compact for these values of *k*, we will incur some overhead from querying it more often. For completeness, we also show the corresponding density performance plot in Figure 11 (left), which confirms that our model converges well even for large *k*.

## 5. CONCLUSION

We introduce a novel framework called DeepMinimizer for learning *sequence-specific* minimizers. This is achieved via casting minimizer selection as optimizing a *k*-mer scoring function ρ. We propose a more well-behaved search space for minimizers, given by a neural network parameterization of ρ, called PriorityNet. Then, we introduce a complementary network, called TemplateNet, which pinpoints optimal scoring templates and guides PriorityNet to the neighborhood of low-density assignments around them.

Coupling these networks leads to a fully differentiable proxy objective that can effectively leverage gradient-based learning techniques. DeepMinimizer obtains better performance than state-of-the-art sequence-agnostic and sequence-aware minimizer selection schemes, especially on known hard tasks such as sketching the repetitive centromere region of Chromosome X.
